# Developmental regulation of olfactory circuit formation in mice

**DOI:** 10.1111/dgd.12657

**Published:** 2020-02-28

**Authors:** Hitoshi Sakano

**Affiliations:** ^1^ Department of Brain Function School of Medical Sciences University of Fukui Fukui Japan

**Keywords:** amygdala, axon guidance, decision making, imprinting, olfactory circuit

## Abstract

In mammals, odorants induce various behavioral responses that are critical to the survival of the individual and species. Binding signals of odorants to odorant receptors (ORs) expressed in the olfactory epithelia are converted to an odor map, a pattern of activated glomeruli, in the olfactory bulb (OB). This topographic map is used to identify odorants for memory‐based learned decisions. In the embryo, a coarse olfactory map is generated in the OB by a combination of dorsal‐ventral and anterior‐posterior targeting of olfactory sensory neurons (OSNs), using specific sets of axon‐guidance molecules. During the process of OSN projection, odor signals are sorted into distinct odor qualities in separate functional domains in the OB. Odor information is then conveyed by the projection neurons, mitral/tufted cells, to various regions in the olfactory cortex, particularly to the amygdala for innate olfactory decisions. Although the basic architecture of hard‐wired circuits is generated by a genetic program, innate olfactory responses are modified by neonatal odor experience in an activity‐dependent manner. Stimulus‐driven OR activity promotes post‐synaptic events and dendrite selection in the responding glomeruli making them larger. As a result, enhanced odor inputs in neonates establish imprinted olfactory memory that induces attractive responses in adults, even when the odor quality is innately aversive. In this paper, I will provide an overview of the recent progress made in the olfactory circuit formation in mice.

## Graphical abstract

Binding signals of odorants detected in the olfactory epithelium (OE) are transmitted to the olfactory bulb (OB) and converted to a topographic map of activated glomeruli. Odor information is then conveyed by projection neurons to the olfactory cortex (OC) for making decisions of the odor quality to mediate behavioral outputs. Decisions are made via two distinct neural circuits; one is innate and the other is learned pathway.

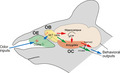

## 1 INTRODUCTION

In mammals, the olfactory system plays an important role in inducing various responses for their survival, for example, attraction, aversion, fear, and social behaviors. In the mouse, odorants are detected by olfactory sensory neurons (OSNs) in the olfactory epithelium (OE) using more than 1,000 different odorant‐receptor (OR) species (Buck & Axel, 1991). As seen in the immune system for allelic exclusion of antibody genes, each OSN expresses only one functional *OR* gene in a mono‐allelic manner, which is referred to as the “one neuron‐one receptor rule” (Serizawa et al., 2003). Furthermore, OSNs expressing the same OR species converge their axons to a stereotyped location in the olfactory bulb (OB) forming a glomerular structure (Mombaerts et al., 1996). This is another rule, one glomerulus‐one receptor, for OSN projection. As a single odorant can interact with multiple OR species with varying magnitudes (Malnic, Hirono, Sato, & Buck, 1999), odor information detected in the OE is topographically represented in the OB as the pattern of activated glomeruli, an odor map (Mori & Sakano, 2011).

During the process of primary projection of OSN axons, olfactory information is sorted into distinct odor qualities in separate OB regions. For example, fox odor and spoiled food smell are processed by the glomeruli located in the dorsal OB for aversive responses (Kobayakawa et al., 2007). In contrast, glomeruli responsive to attractive social cues are clustered in the posterodorsal OB (Inokuchi et al., 2017). It has been shown that photo‐stimulation of mitral/tufted (M/T) cells in a particular functional domain in the OB can elicit innate odor responses even through single glomerular species (Saito et al., 2017). Thus, the glomerular map plays dual roles in processing olfactory signals by serving as a projection screen for pattern recognition of odor information and by providing functional domains for innate olfactory decisions. Like abbreviation dialing with a push‐phone, the glomerular map contains key glomeruli that can induce specific innate responses.

**Figure 1:**
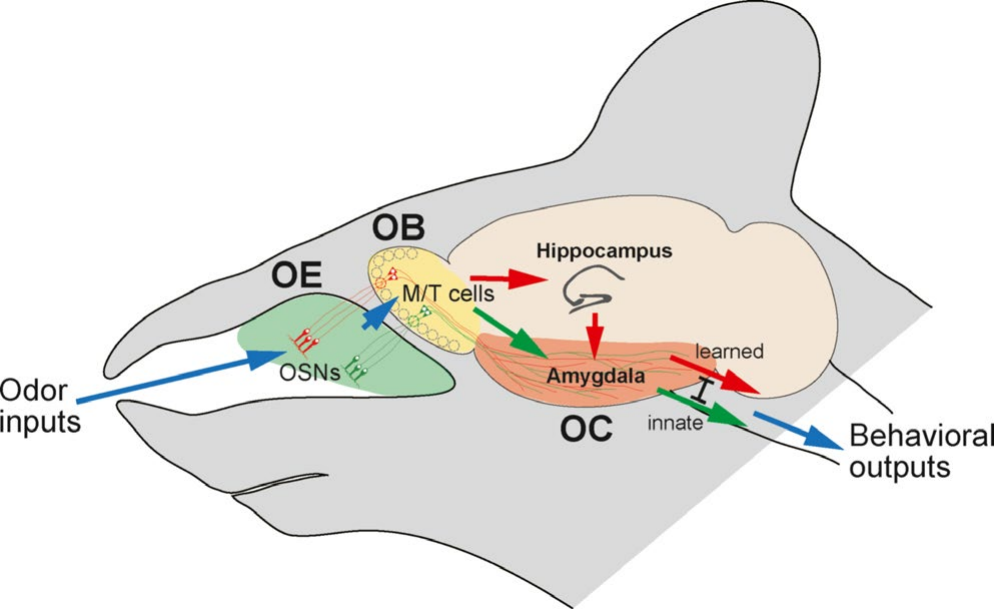
Mouse olfactory circuits. Odorants are detected by OR molecules expressed in OSNs within the OE. Binding signals of odorants are converted to a topographic map of activated glomeruli in the OB. Odor information is then conveyed by M/T cells to various regions in the OC for odor recognition and decision making

For memory‐based odor recognition, topographic information of an odor map in the OB is then distributed to various OC regions by the projection neurons, M/T cells (Igarashi et al., 2012). Odor information is also transmitted from the functional domains directly to the amygdala by mitral cells (MCs) to elicit innate odor responses (Inokuchi et al., 2017; Miyamichi et al., 2011; Root, Denny, Hen, & Axel, 2014) (Figure 1). How are the axons of M/T cells guided to specific OC regions and how are their dendrites synapsed with partner glomeruli to transmit odor signals properly? In this review article, we will overview the developmental regulation of olfactory map formation and summarize the recent progress in the study of olfactory circuit formation for decision making in mice.

## 2 PRIMARY PROJECTION

A remarkable feature of OSN projection is that OR molecules play an instructive role in projecting OSN axons to the OB. Since OR molecules can be detected in axon termini (Barnea et al., 2004; Feinstein & Mombaerts, 2004), it has been suggested that the OR protein itself may recognize guidance cues in the OB and also mediate homophilic interactions of similar OSN axons for glomerular sorting (Mombaerts, 2006). However, recent studies indicate that instead of directly acting as axon‐guidance receptors and sorting molecules, ORs regulate transcription levels of axon‐guidance and axon‐sorting molecules using cyclic adenosine monophosphate (cAMP) as a second messenger (Imai, Suzuki, & Sakano, 2006; Sakano, 2010).

For dorsal‐ventral (D‐V) targeting, positional information of OSNs within the OE regulates both *OR* gene choice and expression levels of axon‐guidance molecules, thus correlating the OR species to their glomerular locations (Miyamichi, Serizawa, Kimura, & Sakano, 2005; Takeuchi et al., 2010). In contrast, anterior‐posterior (A‐P) targeting is directly instructed by OR molecules. Spontaneous receptor activity of ORs regulates the transcription of A‐P targeting genes via cAMP whose levels are uniquely determined by the expressed OR molecules (Nakashima et al., 2013). OR‐derived cAMP also regulates the transcriptional levels of axon‐sorting molecules for glomerular segregation (Serizawa et al., 2006). However, unlike A‐P targeting, glomerular segregation is regulated by the neuronal activity of OSNs. How do the OR molecules differentially regulate both A‐P targeting and glomerular segregation using cAMP as a second messenger? What are the sources of cAMP, and how do the signals differentially regulate these two processes? Recent studies have unveiled these questions by using mutant mice for axon‐guidance and signaling molecules.

### 2.1 Projection along the A‐P axis

**Figure 2:**
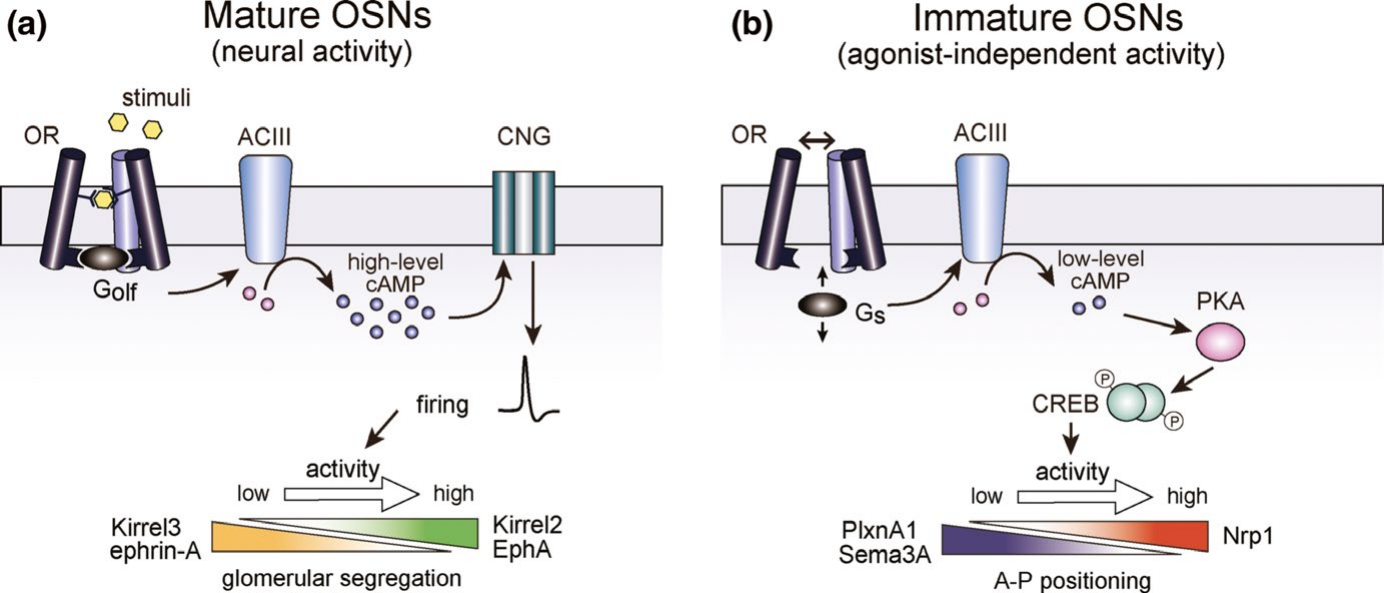
Two distinct types of OR‐specific activities. (a) Canonical signal‐transduction pathway. Glomerular segregation is regulated by stimulus‐driven neuronal activity in mature OSNs, using G_olf_ and CNG channels. (b) Non‐canonical signal‐transduction pathway. A‐P targeting is regulated by agonist‐independent receptor activity of ORs in immature OSNs with G_s_ and protein kinase PKA. Both pathways utilize adenylyl cyclase type III (ACIII) for cAMP production. OR‐specific cAMP determines transcription levels of A‐P targeting or glomerular segregation molecules. Figures are modified from Nakashima et al. (2013)

In mature OSNs, binding signals of odorants are converted into neuronal activity via cAMP. The olfactory‐specific G‐protein (G_olf_) activates adenylyl cyclase type III (ACIII) and generates cAMP that opens cyclic‐nucleotide gated (CNG) channels (Wong et al., 2000). CNG channels, together with chloride channels (Stephan et al., 2009), depolarize the plasma membrane, thus generating the action potential (Figure 2a). Targeted knockouts (KOs) of G_olf_ and CNG‐A2, a component of CNG channels, cause severe anosmia (Brunet, Gold, & Ngai, 1996). Curiously, however, these KOs do not demonstrate major defects in axonal projection of OSNs (Belluscio, Gold, Nemes, & Axel, 1998; Lin et al., 2000; Zheng, Feinstein, Bozza, Rodriguez, & Mombaerts, 2000). Therefore, it was initially thought that OR‐derived cAMP signals were not used for OSN projection.

Although the KOs of G_olf_ and CNG‐A2 did not perturb axon targeting of OSNs, the KO of ACIII demonstrated severe defects in olfactory map formation (Chesler et al., 2007; Col, Matsuo, Storm, & Rodoriguez, 2007). Thus, it was assumed that OR‐instructed OSN projection uses an alternate G‐protein and does not require CNG‐channel activity. To examine this possibility, Imai et al. (2006) generated a mutant OR whose G‐protein coupling motif, DRY, was mutated to RDY. The RDY mutant can bind its odor ligand but is incapable of generating cAMP. OSN axons expressing this mutant OR remain in the anterior OB and fail to converge to a specific glomerulus. These defects are restored by co‐expression of the constitutively‐active (ca) mutant of stimulus G‐protein (G_s_), caG_s_. Partial rescue is also observed with the ca mutant of protein kinase PKA. Thus, PKA‐mediated transcriptional regulation appears to be involved in A‐P projection, using G_s_ without involving G_olf_ and CNG channels (Figure 2b). Interestingly, caG_s_ results in a posterior shift of glomeruli when expressed with the wild‐type (WT) OR, whereas dominant‐negative PKA results in an anterior shift (Imai et al., 2006). These findings demonstrate that it is the OR‐derived cAMP signals, rather than the direct action of OR molecules, which determine the target destination of OSNs along the A‐P axis in the OB.

### 2.2 Pre‐target axon sorting for A‐P projection

How do the cAMP signals regulate A‐P targeting? As aforementioned, OSNs that produce higher levels of cAMP project their axons to the posterior OB, whereas those producing lower levels target the anterior OB. In OSN axons, an axon‐guidance receptor Neuropilin 1 (Nrp1) is transcriptionally regulated by cAMP and found in an anterior^low^/posterior^high^ gradient in the OB. Increases and decreases of Nrp1 expression in OSNs cause posterior and anterior shifts of corresponding glomeruli, respectively (Imai et al., 2006). Then, how does the axon‐guidance receptor Nrp1 regulate the topographic order of the olfactory map? Sperry (1963) proposed the “chemo‐affinity model” for axonal projection, in which target cells present chemical cues to guide axons to their destination. Since then, it has been generally thought that the topography of the neural map is determined by interactions between guidance receptors expressed at axon termini and positional cues present at the target.

In contrast to the Sperry model, OSN axons are able to converge and form loci even in the absence of the target OB (Bulfone et al., 1998; St John, Clarris, McKeown, Royal, & Key, 2003). Interestingly, map order emerges in axon bundles, well before they reach the target (Satoda, Takagi, Ohta, Hirata, & Fujisawa, 1995). Imai et al. (2009) found that pre‐target axon sorting of OSNs plays an important role in organizing of the olfactory map topography. Within the axon bundles of OSNs, Nrp1 and its repulsive ligand Semaphorin 3A (Sema3A) are expressed in a complementary manner. Furthermore, Nrp1^low^/Sema3A^high^ axons are sorted to the central compartment of the bundle, whereas Nrp1^high^/Sema3A^low^ axons are confined to the outer‐lateral compartment. OSN‐specific KO of Nrp1 or Sema3A not only perturbs axon sorting within the bundle but also causes shifts of glomeruli along the A‐P axis. These results demonstrate that pre‐target axon sorting within bundles contributes to establish olfactory map topography.

### 2.3 Projection along the D‐V axis

For OSN projection along the D‐V axis, there is a close correlation between the anatomical locations of OSNs in the OE and their projection sites in the OB (Astic, Saucier, & Holley, 1987). The preservation of spatial relationships of neuronal cell bodies and their axonal projection sites is widely seen in other brain regions (Luo & Flanagan, 2007; McLaughlin & O’Leary, 2005). In the mouse olfactory system, two sets of repulsive signaling molecules, Nrp2/Sema3F and Robo2/Slit1, are known to participate in D‐V projection. Dorsal (D)‐zone OSN axons that are Robo2‐positive (Robo2^+^) navigate to the D domain of the OB through the repulsive interactions with its ligand Slit1 expressed in the ventral (V) domain of the OB (Cho, Lepine, Andrews, Parnavelas, & Cloutier, 2007). Robo1 is also involved in guiding D‐zone OSN axons (Aoki, Takeuchi, Nakashima, Nishizumi, & Sakano, 2013). These guidance molecules contribute to the separation of D and V domains in the OB (Takeuchi et al., 2010).

How is the positional information of OSNs in the OE translated to their target sites during olfactory map formation? Takeuchi et al. (2010) found that Nrp2 and its repulsive ligand Sema3F are both expressed by OSN axons in a complementary manner to regulate D‐V projection. Although expression levels of D‐V guidance molecules, Nrp2 and Sema3F, are closely correlated with the expressed OR species, the transcription of their genes is not downstream of OR signaling. It is assumed that both *OR* gene choice and expression levels of D‐V projection molecules are commonly regulated by positional information within the OE, thus correlating the transcriptional levels of guidance genes with the expressed OR species.

**Figure 3:**
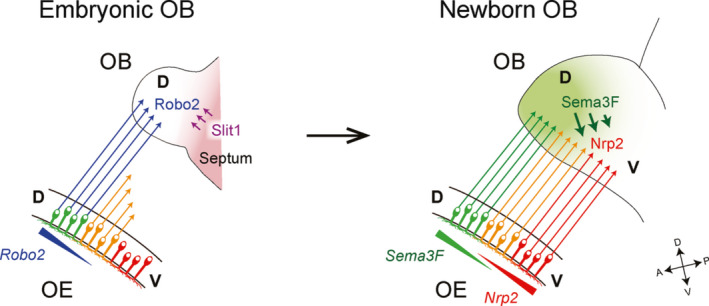
D‐V targeting of OSN axons. Early‐arriving D‐region axons express Robo2 and project to the anterodorsal OB with the aid of repulsive interactions with Slit1 (left). Late‐arriving V‐region axons are Nrp2‐positive and ventrally repelled by Sema3F secreted by the dorsal OSN axons (right). Sequential arrival of OSN axons and graded/complementary expression of Nrp2 and Sema3F help establish the topographic order along the D‐V axis. Figures are modified from Takeuchi et al. (2010)

During development, OSNs in the D‐region mature earlier than those in the V‐region. The D‐region OSNs target their axons to the embryonic OB before the V‐region axons arrive (Sullivan, Bohm, Ressler, Horowitz, & Buck, 1995; Takeuchi et al., 2010). This observation points toward a possibility that the repulsive ligand Sema3F, produced by early‐arriving D‐region axons, is deposited in the anterodorsal OB to serve as a guidance cue to repel late‐arriving V‐region axons that express Nrp2 (Figure 3). Then, what guides pioneer OSN axons to the anterodorsal area acting as a landmark in the OB? Robo2^+^ D‐region axons are guided to the D domain of the OB by repulsive interactions with a Robo2 ligand, Slit1 (Cho et al., 2007; Nguyen‐Ba‐Charvet, Di Meglio, Fouquet, & Chedotal, 2008). During development, the glomerular map expands ventrally and axonal projection of OSNs occurs sequentially from the dorsomedial to the ventrolateral regions in the OE. This sequential arrival of OSN axons helps establish the topographic map order along the D‐V axis in the OB.

### 2.4 Activity‐dependent glomerular segregation

During embryonic development, an olfactory map topography is established based on a genetic program that is independent of the neuronal activity. After OSN axons reach their approximate destinations in the OB, refinement of the glomerular map needs to occur through fasciculation and segregation of OSN axons in an activity‐dependent manner.

**Figure 4:**
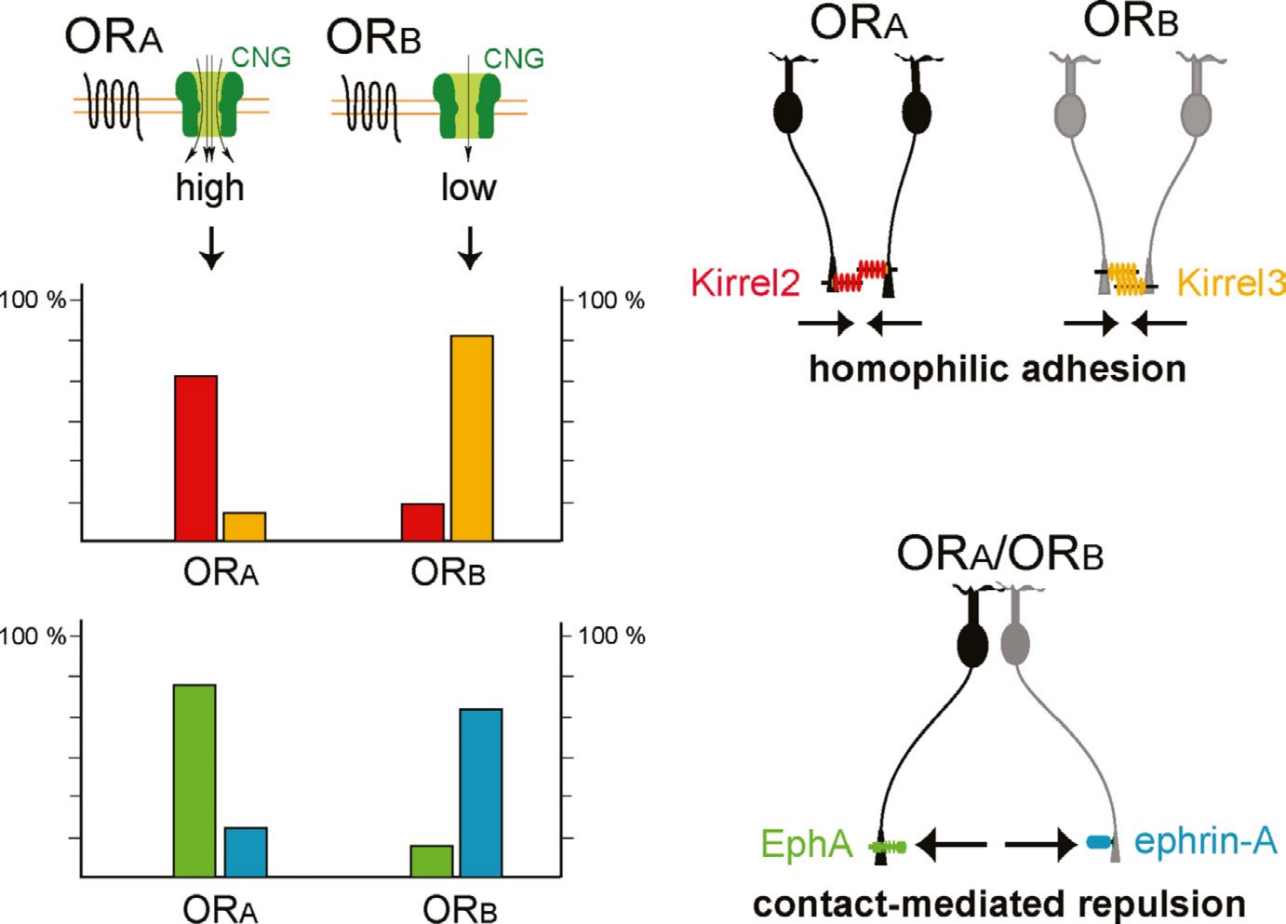
Activity‐dependent glomerular map refinement. Two sets of axon‐sorting molecules are involved in glomerular segregation. One set is hemophilic adhesion molecules, for example, Kirrel2 and Kirrel3, and the other is repulsion molecules, for example, ephrin‐A and EphA receptors. Activity‐high ORs (OR_A_) produce higher levels of Kirrel2 and ephrin‐A, and lower levels of Kirrel3 and ephrin‐A. In contrast, activity‐low ORs (OR_B_) produce these molecules in an opposite manner. OSNs expressing the same type of OR fasciculate their axons by hemophilic interactions with Kirrel2 or Kirrel3, whereas those expressing different types of ORs are separated by contact‐mediated repulsive interactions of ephrin‐A ligand and EphA receptor. Figures are modified from Serizawa et al. (2006)

To study how the glomerular segregation is regulated by OR molecules, Serizawa et al. (2006) searched for a group of genes whose expression profiles correlated with the OR species. Using a transgenic (Tg) mouse in which the majority of OSNs express a particular OR, such genes were identified. They include ones that code for homophilic adhesive molecules, Kirrel2 and Kirrel3, that are expressed in a complementary manner. Repulsive molecules, such as EphA receptors and ephrin‐A ligands, are also identified in OSN axons in a complementary manner. A specific set of adhesive and repulsive molecules, whose expression levels are determined by ORs, appears to regulate the axonal fasciculation of OSNs (Figure 4). Repulsive interactions between the two subsets of OSN axons, one that is ephrin‐A^high^/EphA^low^ and the other that is ephrin‐A^low^/EphA^high^, are important for the segregation of dissimilar axons. Homophilic adhesive interactions within the two subsets of axons, Kirrel2^high^/Kirrel3^low^ and Kirrel2^low^/Kirrel3^high^, mediate bundling of similar OSN axons.

Using the optogenetic method in the cultured cells, Nakashima et al. (2019) reported that temporal spike patterns of spontaneous activity may regulate glomerular segregation. In the dissociated OSNs, intrinsic neuronal activity has been detected without odor ligands (Reisert, 2010). In the visual system, spontaneous firing‐waves in the retina are known to play a critical role in shaping the neural map in neonates (Feller, 2002). It is interesting to know whether a similar intrinsic activity is involved in the olfactory map formation. If the temporal spike pattern determines the expression levels of glomerular segregation molecules, how is the firing pattern read out and differentially translated into the transcriptional signals of axon‐sorting molecules? It is also important to determine whether OR activity required for glomerular segregation is entirely intrinsic or odor‐evoked.

It should be noted that an olfactory map generated during embryonic development is a continuous map whose topography is established by graded and complimentary distributions of axon‐guidance receptors and their repulsive ligands. This continuous map is further converted to a discrete map of glomeruli. Activity‐dependent axon sorting contributes not only to map refinement, but also to the conversion of the olfactory map from continuous to discrete by segregating glomeruli (Yu et al., 2004; Zhao & Reed, 2001). This is in sharp contrast to the visual system where the map remains continuous (Luo & Flanagan, 2007).

### 2.5 Agonist‐independent OR activities

**Figure 5:**
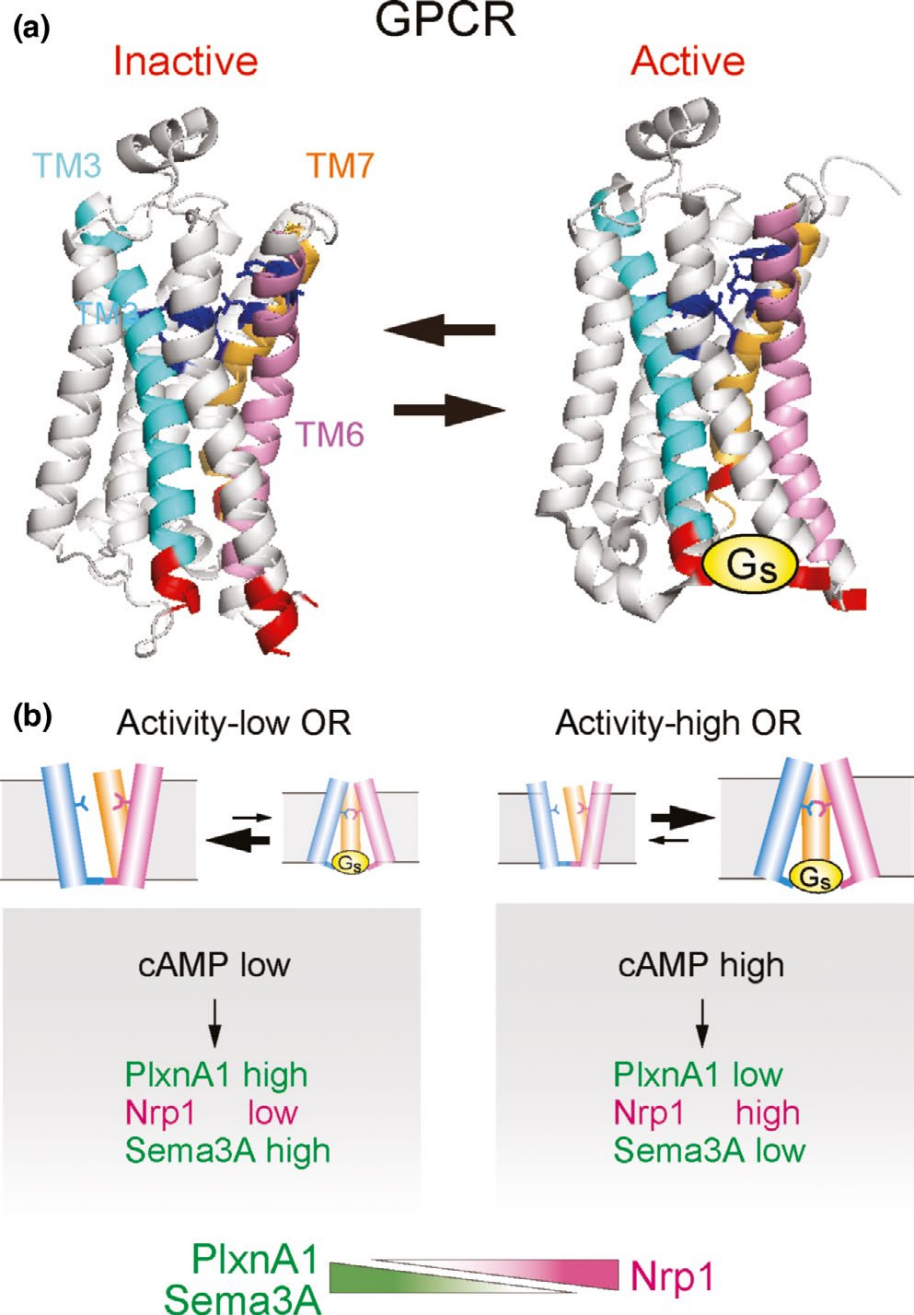
Baseline activity of ORs. (a) Spontaneous transition of GPCR conformation. In the absence of agonist and inverse agonist, GPCRs generate baseline activity with G_s_ by conformational interchange between the active and inactive forms. Three‐dimensional structures of β2‐AR are modified from Rasmussen et al. (2011). (b) Regulation of A‐P targeting by OR‐specific baseline activity. Each OR possesses a unique level of receptor activity that determines transcription levels of A‐P targeting molecules, for example, Nrp1 and PlxnA1, using cAMP as a second messenger. Figures are modified from Nakashima et al. (2013)

Unlike for local sorting of OSN axons, OR‐derived signals for global targeting are not stimulus‐driven. Then, what kind of OR activity could be responsible for A‐P projection, and how is it generated? G‐protein‐coupled receptors (GPCRs), including ORs, are known to possess two conformational states, active and inactive (Kobilka & Deupi, 2007). By spontaneously flipping between the two different conformations, GPCRs produce baseline levels of cAMP in the absence of agonists and inverse agonists (Figure 5). For different OR species, variable but specific levels of baseline activities can be detected (Nakashima et al., 2013). This agonist‐independent GPCR activity had long been considered to be noise, and its functional role was not fully appreciated.

To examine whether the agonist‐independent baseline activity is responsible for regulating A‐P targeting, various mutants of the β2‐adrenergic receptor (β2‐AR) have proven useful. The β2‐AR is a GPCR with the highest sequence homology to ORs. When expressed in OSNs with the *OR* gene promoter, the β2‐AR maintains the one neuron‐one receptor rule, couples with the α subunit of G_s_ or G_olf_, and substitutes ORs for receptor‐instructed axonal projection (Feinstein, Bozza, Rodriguez, Vassalli, & Mombaerts, 2004). Based on mutational studies, the key amino‐acid residues are well characterized in the β2‐AR for G‐protein coupling, ligand binding, and generation of agonist‐independent activity (Ballesteros et al., 2001; O’Dowd et al., 1988; Savarese & Fraser, 1992). Furthermore, the three‐dimensional structures of β2‐AR with G_s_ are known (Rasmussen et al., 2011). As a result of these favorable features, Nakashima et al. (2013) utilized β2‐AR mutants for the transgenic analysis of the agonist‐independent GPCR activity in axonal projection of OSNs.

Among the β2‐AR mutants, those that affect conformational transitions demonstrate altered agonist‐independent baseline activity. In the Tg mice expressing both mutant and WT β*2‐AR* genes, the activity‐low and activity‐high β2‐AR mutants generate glomeruli anterior and posterior to those of the WT, respectively (Nakashima et al., 2013). Nrp1 levels increase in the OSNs expressing the activity‐high β2‐AR mutants, but are lowered by the activity‐low mutation. The results are inverse for another A‐P targeting molecule, Plexin Al (PlxnA1), because its expression is inversely regulated by cAMP signals. It is notable that levels of glomerular segregation molecules, for example, Kirrel2 and Kirrel3, are not affected by the mutations of baseline activity. These results demonstrate that it is the agonist‐independent OR activity that determines the transcriptional levels of A‐P targeting molecules.

### 2.6 Differential regulation of targeting and sorting of OSN axons

As aforementioned, OR‐instructed A‐P targeting and glomerular segregation are differentially regulated in OSNs by two distinct OR‐derived cAMP signals. How are these two types of regulation separately controlled in OSN cells, despite both being instructed by the same OR identity? It was found that the two types of OR‐derived signals are transduced at different stages of olfactory development: A‐P targeting occurs in immature OSNs and glomerular segregation in mature OSNs. The differences are also due to the subcellular localization of ORs, i.e., axon termini for targeting versus cilia for odor detection.

Although such spatial and temporal insulation of the distinct signals may explain the differential regulation, the major basis for the difference is a result of the distinct sources of cAMP using different G‐proteins, G_s_ and G_olf_ (Nakashima et al., 2013) In vitro experiments using fusion GPCR proteins with G_s_ or G_olf_ demonstrate that G_s_ efficiently detect agonist‐independent receptor activity, whereas G_olf_ is adapted to precisely respond to temporal changes in ligand concentrations. In the cKO of G_s_, A‐P targeting is perturbed, but glomerular segregation is not. In contrast, cKO of G_olf_ affects glomerular segregation, but not A‐P targeting. A‐P targeting and glomerular segregation, both using OR‐derived cAMP, are separately regulated by the non‐canonical and canonical signal‐transduction pathways, respectively, using separate G‐proteins at different stages of OSN development (Figure 2).

## 3 SECONDARY PROJECTION

**Figure 6:**
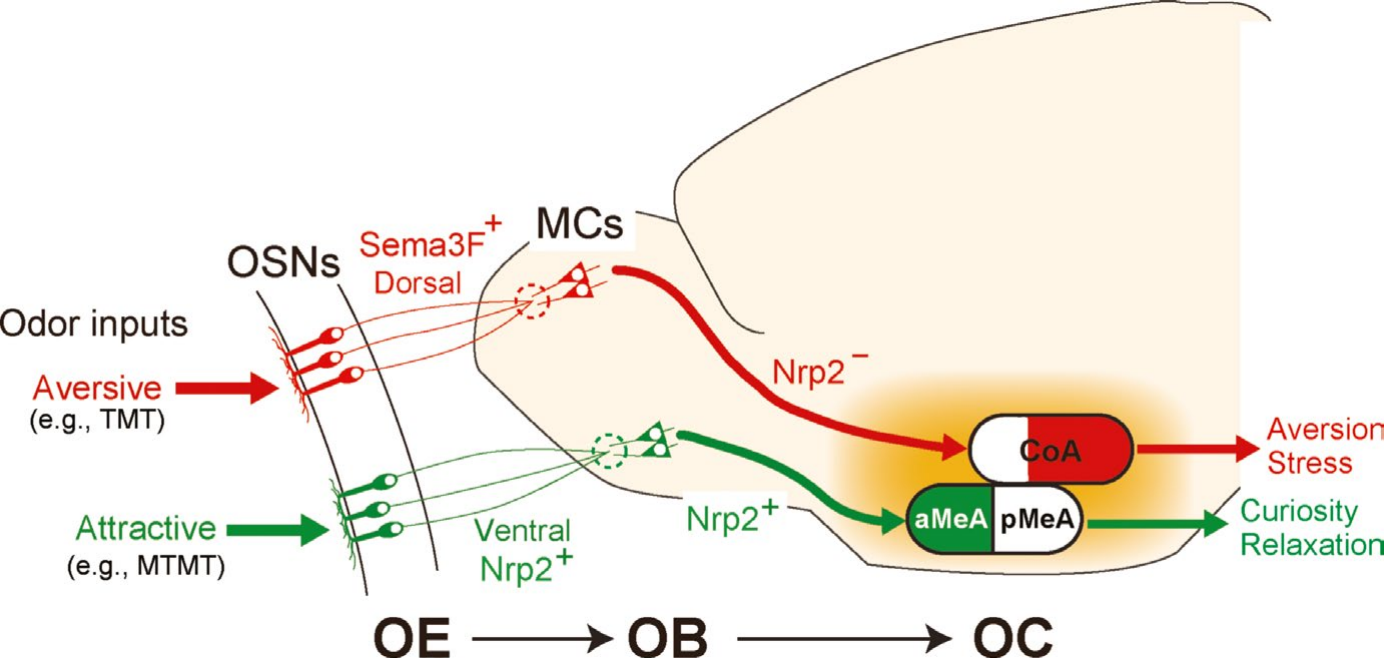
Hard‐wired neural circuits that mediate innate odor responses. Odor information detected in the OE is roughly sorted into two different qualities, aversive and attractive, along the D‐V axis during the process of primary projection. Attractive social information (e.g., male urine odor MTMT) is collected by Nrp2^+^ OSN axons in the posterodorsal OB and is further transmitted to the anterior MeA by Nrp2^+^ MCs. In contrast, aversive information (e.g., fox smell TMT) assembled by Nrp2^‐^ OSNs in the posterodorsal OB is forwarded to the posteromedial CoA

During the process of primary projection from the OE to the OB, odor information is roughly sorted into two distinct qualities along the D‐V axis, one that is aversive in the dorsal OB and the other that is attractive in the posteroventral OB (Figure 6). Odor signals are then transmitted to various areas in the OC by M/T cells. In the amygdala, the posteromedial region of the cortical amygdala (CoA) receive the aversive odor information from the posterodorsal OB to mediate innate avoidance responses (Maras & Petrulis, 2006; Root et al., 2014). In contrast, for the attractive social cues, responding glomeruli are clustered in the posteroventral OB (Lin, Zhang, Block, & Katz, 2005; Yoshikawa, Nakagawa, Mori, Watanabe, & Touhara, 2013), whose information is transmitted to the anterior region of the medial amygdala (MeA) to mediate attractive social responses (Lehman, Winans, & Powers, 1980).

How is it then that the M/T cells send their axons correctly to the specific OC regions to elicit proper odor responses? In contrast to the primary projection of OSNs, not much is known about the secondary projection. The main reason that makes these studies difficult has been the lack of known subset markers essential for generating the conditional knockouts (cKOs) for M/T‐cell projection. Recently, it was found that Nrp2 can be used for the study of migration and projection of mitral cells (MCs) in mice (Inokuchi et al., 2017).

### 3.1 Migration and segregation of MCs within the OB

Nrp2 and its repulsive ligand Sema3F have previously been reported as axon‐guidance molecules that regulate primary projection of OSNs along the D‐V axis (Takeuchi et al., 2010). Like in the glomerular layer for OSN axons, Nrp2 levels are high in the V‐region, but low in the D‐region in the mitral‐cell layer (MCL). During embryonic development, MC precursors are born in the ventricular zone within the OB and migrate radially to the MCL (Imamura, Ayoub, Rakic, & Greer, 2011). Nrp2^+^ MCs further migrate tangentially to the ventral OB as the OB ventrally expands, whereas Nrp2^−^ MCs remain in the embryonic OB region that represents the dorsal OB in adults. Since MC dendrites synapse with OSN axons in the nearest neighboring glomeruli (Nishizumi et al., 2019), D/V partitioning provides a topographical and functional separation of MCs within the OB.

Then, what mediates the segregation and migration of Nrp2^+^ and Nrp2^−^ MCs? In the total KO of Nrp2, both OSN projection and MC segregation are perturbed along the D‐V axis (Inokuchi et al., 2017). However, in the OSN‐specific or MC‐specific cKO of Nrp2, either the OSN projection or MC migration is affected, but not both. This observation excludes a possibility of Nrp2‐mediated cross‐talk between the OSN axons and MCs for parallel guidance. Interestingly, in the OSN‐specific cKO of Sema3F, not only OSN targeting but also MC migration is perturbed, indicating that Sema3F secreted by the D‐region OSN axons guides both Nrp2^+^ OSN axons and Nrp2^+^ MCs to the posteroventral OB (Inokuchi et al., 2017). This co‐regulation is important for functional pairing of their partner MC primary dendrites with glomeruli.

### 3.2 Matching of MCs with partner glomeruli

**Figure 7:**
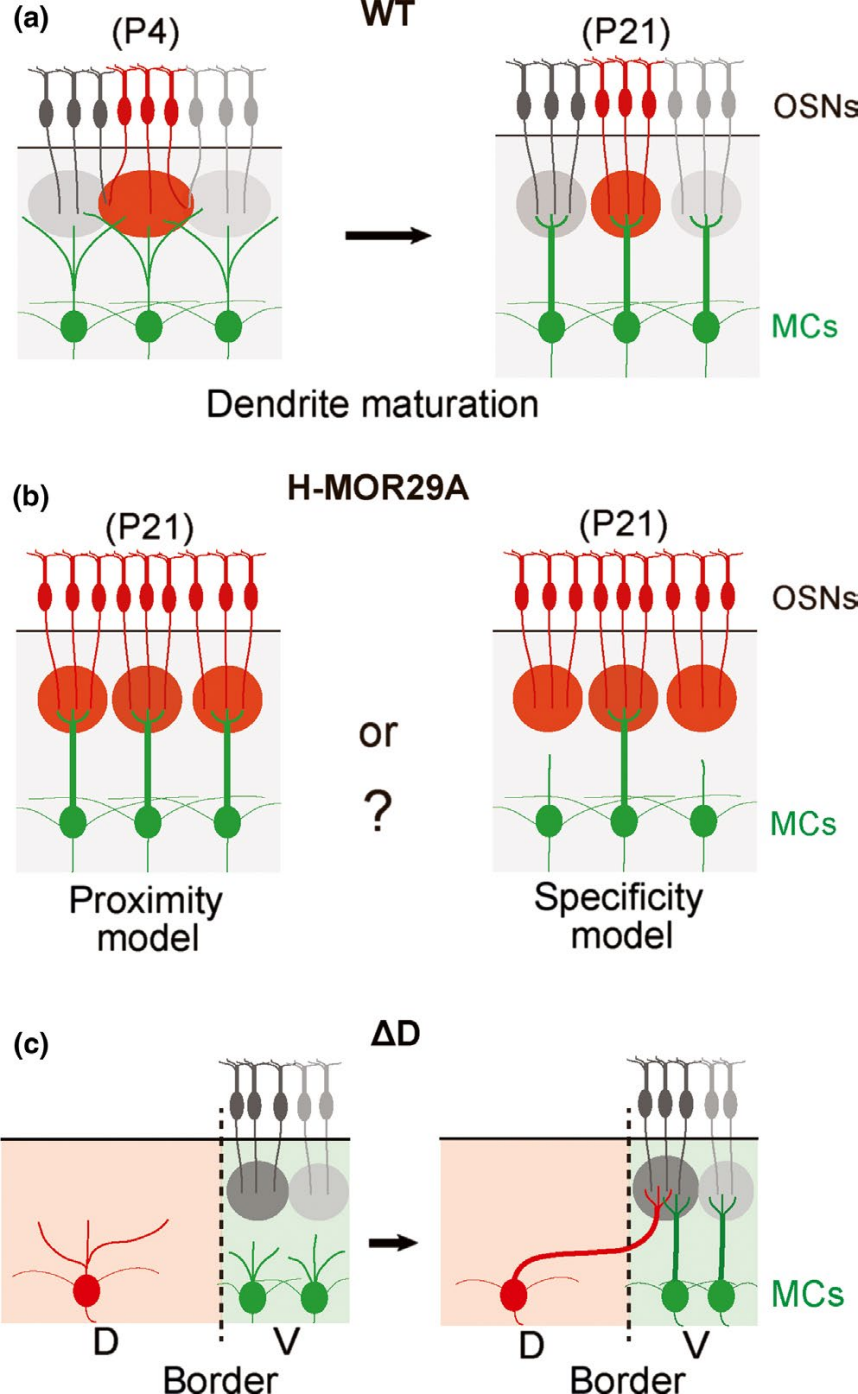
Matching of MCs with partner glomeruli. (a) Maturation of MC dendrites. During development, immature MCs extend multiple dendrites to neighboring glomeruli (at P4). At the mature stage (P21), only one dendrite can survive to become a primary dendrite that synapses with OSN axons within the partner glomerulus. (b) Partner finding and dendrite selection of MCs. In the H‐MOR29A mice, transgenic MOR29A glomeruli are clustered surrounding the endogenous MOR29A glomerulus. In such an unusual situation, how do the MCs contacting the Tg‐MOR29A glomeruli behave for primary dendrite selection? One possibility is that matching of MCs with partner glomeruli takes place based on the OR specificity of glomeruli (right). The other possibility is that MCs extend their primary dendrite to the nearest neighboring glomerulus based on the proximity of distance (left). (c). Partner finding of MCs in the △D mutant. In the △D, glomeruli are specifically ablated by diphtheria toxin in the D domain of the OB. In this mutant mouse, MCs in the D domain extend their primary dendrite to the V‐domain glomerulus crossing over the D‐V border of the OB

In order to mediate innate odor responses, proper pairing is required between the OSN axons and MC primary dendrites. During development, MCs initially possess multiple dendrites extending to neighboring glomeruli. However, after selecting the partner glomerulus, only one dendrite connecting to it can survive and mature to the primary dendrite (Figure 7a). How are the MCs able to find the right partner to initiate synapse formation? One possibility is that OSN axons and MC dendrites recognize the partners’ identity when the matching takes place. If this is the case, the identity of OSNs is likely established by the expressed OR species. This then engenders the question of the identity of MCs and how it is recognized by OSN axons. Another possibility is that there is no such molecular code for MCs to be recognized by OSN axons. MC dendrites may simply find their partner OSN axons based on the physical proximity without regard to OR specificity and connect to the nearest neighboring glomeruli. In this case, it is important for MCs to migrate to appropriate locations in the OB before synapsing with OSN axons.

To address what mediates proper matching within the glomeruli, Nishizumi et al. (2019) studied mutant mice in which the glomerular map formation is perturbed. First, they analyzed the H‐MOR29A mouse where multiple glomeruli for the Tg MOR29A are clustered surrounding the endogenous MOR29A glomerulus (Figure 7b). In the H mouse, the frequency of the choice for a particular *OR* gene is largely increased by the attached *H* enhancer (Nishizumi, Kumasaka, Inoue, Nakashima, & Sakano, 2007; Serizawa et al., 2003). It should be noted that in the WT situation, MCs underneath the clustered Tg MOR29A glomeruli are supposed to pair with OSN axons whose OR identities are different from MOR29A (Figure 7b). Interestingly, in the H‐MOR29A mouse, no abnormality of dendrite selection is observed for the MCs connecting to the ectopic Tg glomeruli. This observation indicates that MC dendrites find their partners in the nearest neighboring region based on the physical distance without regarding the OR identity.

Another mouse analyzed was the △D mutant where the glomeruli are specifically ablated in the dorsal OB by diphtheria toxin (Kobayakawa et al., 2007). Near the D/V border, D‐region MCs extend their dendrites to the V‐region glomeruli, crossing over the D/V border (Figure 7c). It appears that primary dendrites of MCs can synapse with OSN axons in the nearest neighboring glomerulus regardless of its OR specificity.

### 3.3 Targeting of MCs to the amygdala

M/T cells are bipolar neurons, synapsing with OSN axons and targeting to specific areas in the OC. After migrating to appropriate locations in the MCL, MCs extend their primary dendrites to the partner glomeruli and also send their axons to various brain regions along the lateral olfactory tract. By using the Tg mouse in which Nrp2^+^ MCs express fluorescence protein, EYFP, the trajectory of axonal projection can be traced. In this mouse, the Nrp2^+^ posteroventral MCs send their axons to the anterior MeA, but not the Nrp2^‐^ dorsal MCs that are positive for OCAM, a dorsal MC marker. In the MC‐specific cKO of Nrp2, MC projection to the anterior MeA is perturbed. These observations indicate a possible circuit link between the posteroventral OB and the anterior MeA via Nrp2^+^ MCs. This connection can be directly examined by trans‐synaptic labeling with the rabies virus that transmits across synapses in a retrograde and monosynaptic manner. Rabies virus injected into the anterior MeA is indeed detected in the posteroventral OB region, but not in the D‐region OB (Inokuchi et al., 2017).

How are the Nrp2^+^ MC axons correctly guided to the anterior MeA? In the total KO of Sema3F, projection of Nrp2^+^ MCs to the anterior MeA is severely affected, indicating that the repulsive ligand Sema3F is involved in this targeting. Unlike MC migration in the OB, MC projection is not affected in the OSN‐specific cKO of Sema3F, suggesting that the Sema3F in the dorsal OB is not responsible for guiding MC axons to the anterior MeA. Since Sema3F can be detected in the cortical regions surrounding the MeA, Nrp2^+^ MC axons are likely guided to the anterior MeA by repulsive interactions with Sema3F expressed along their trajectory in the embryonic OC. Taken together, Nrp2 plays dual instructive roles through repulsive interactions with Sema3F in regulating migration and targeting of V‐region MCs.

For the study of Nrp2‐mediated MC targeting, Inokuchi et al. (2017) also performed gain‐of‐function experiments by using in utero electroporation. When the *GFP* gene is introduced into the embryonic OB, green signals of GFP are equally distributed in both the D‐ and V‐regions of MCL (Imamura et al., 2011). In contrast, if the human *Nrp2* gene (*hNrp2*) is co‐transfected, GFP^+^ MCs are confined to the V‐region MCL. Furthermore, GFP signals are detected in the anterior MeA in the *hNrp2* transfected mice. Activation of the single axon‐guidance gene *hNrp2 Nrp2* appears to be sufficient to induce not only the migration of MCs to the V‐region MCL but also their axonal projection to the anterior MeA. In this electroporation experiment, the dorsal MC marker OCAM can be detected in the anterior MeA, indicating that ectopic expression of hNrp2 alone can induce MC projection to the anterior MeA even in the dorsal‐lineage MCs that normally send their axons to the CoA.

### 3.4 Functional OB domains for innate odor responses

As already mentioned, the glomerular map is composed of distinct functional domains for different odor qualities. By ablating glomeruli from specific OB regions, Kobayakawa et al. (2007) demonstrated that key glomeruli that elicit fear responses to the fox odor TMT are located in the D_II_ domain of the posterodorsal OB. In contrast, the glomeruli responsive to attractive social cues, for example, methylthio‐methanethiol (MTMT), are confined to the posteroventral OB (Lin et al., 2005).

**Figure 8:**
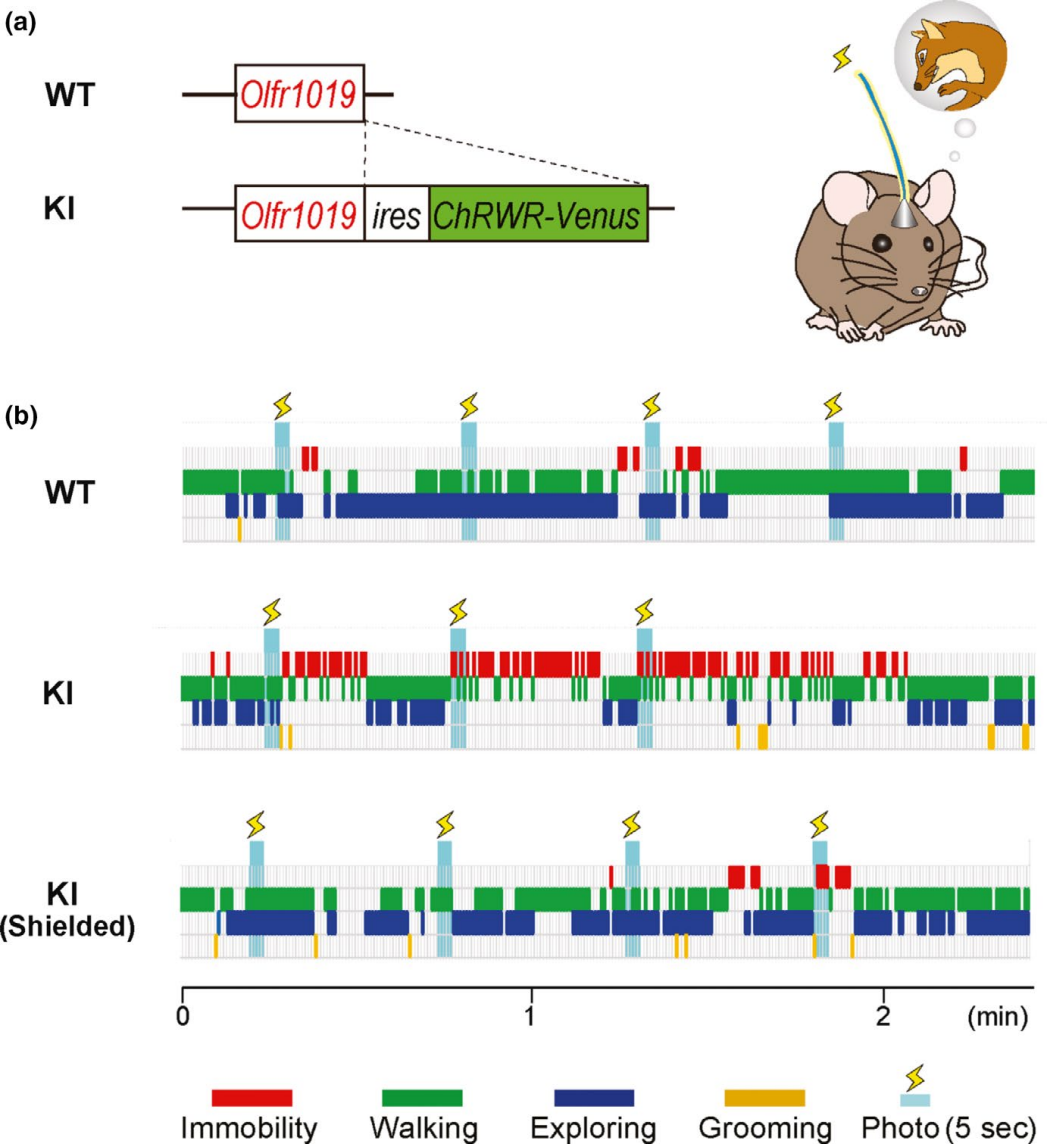
Photo‐activation of a single glomerular species, Olfr1019. (a). A plasmid construct for the knockin (KI) mouse of Olfr1019. The channel rhodopsin gene, *ChRWR*, is introduced into the TMT‐responsive *OR* genes, *Olfr1019*, with the *ires* sequence. For fluorescent labeling of Olfr1019 glomeruli, the *Venus* gene is also inserted. Photo‐activation of the Olfr1019 glomeruli induces immobility (freezing) responses in the KI mouse. (b) Time‐course studies of mouse behaviors. The KI mice are photo‐illuminated and analyzed for their responses. The wild‐type (WT) and aluminum‐foil‐shielded KI are studied in parallel as negative controls. Five‐second illuminations are given every 30 s. Immobility is shown in red. Figures are modified from Saito et al. (2017)

Optogenetic experiments demonstrate that activation of single glomerular species can elicit specific innate odor responses (Figure 8). For example, Saito et al. (2017) found that photo‐activation of one of the TMT‐responsive glomeruli in the D_II_ domain induces freezing (immobility), but not avoidance behavior, indicating that this particular OR, Olfr1019, is specialized for freezing. In the KO of Olfr1019, immobility responses to TMT are reduced, but aversion and stress reactions are not affected. Consistent with this observation, the posteromedial CoA and AmPir that are known to induce aversive responses, are not activated by photo‐illumination of the Olfr1019 glomeruli. These observations demonstrate that TMT‐induced fear may be separated into two different components, immobility and avoidance. Furthermore, the TMT‐induced immobility appears not to be the consequence of stress reactions mediated by a stress hormone, adrenocorticotropic hormone (ACTH).

It is generally thought that an activation pattern of a specific set of glomeruli, an odor map, is transmitted to the OC to discriminate and identify the odorant (Mori & Sakano, 2011). However, the olfactory system uses a different strategy to quickly elicit innate odor responses. Instead of recognizing the pattern of activated glomeruli for the memory‐based decision, a specific innate response can be induced when a particular functional domain in the OB is activated even through single glomerular species. For the odor‐mediated innate responses, there is a close correlation between the functional domains in the OB and specific regions in the amygdala. This is accomplished by gathering glomeruli having the same behavioral quality to a restricted functional domain in the OB and by transmitting odor information correctly to a specific amygdala region. It is important for MCs to ensure that the olfactory inputs with the same odor quality are transmitted to a particular area in the amygdala so that they can induce the same innate outputs.

### 3.5 Nrp2^+^ MCs mediate attractive social responses

For the attractive social responses, a circuit link has been shown to exist between the posteroventral OB and the anterior MeA by dye‐injection and pharmacological experiments (Keller, Douhard, Baum, & Bakker, 2006; Martel & Baum, 2007). The anterior MeA is known to induce innate social behaviors, such as mating and conspecific recognition. It has been thought that social signals come from the vomeronasal organ through the accessary OB that detects non‐volatile pheromones. However, recent studies suggest that the anterior MeA also receives volatile odor inputs from the main OB (Inokuchi et al., 2017; Lehman et al., 1980). Since the anterior MeA is activated by urinary volatiles whose responsive glomeruli are located in the posteroventral OB, Nrp2^+^ MCs likely transmit the attractive social signals to the anterior MeA.

To study the roles of Nrp2^+^ MCs in mediating innate olfactory behaviors, Inokuchi et al. (2017) analyzed the MC‐specific cKO of Nrp2 for odor‐mediated social responses. In the male cKO, ultrasonic vocalization toward females is diminished. In the female cKO, investigation times are significantly lowered towards a male urine volatile, MTMT. Suckling behaviors of pups are also perturbed in the cKO. These results demonstrate that Nrp2^+^ MCs indeed mediate odor‐induced attractive social responses by linking the posteroventral OB and the anterior MeA (Figure 6). This kind of circuit formation that is directed by a distinct set of guidance molecules may be a general rule that is applicable to other innate odor responses. For example, fear to predators’ smell and avoidance to spoiled food odors are induced by glomeruli located in the D_II_ and D_I_ domains in the dorsal OB, respectively. Furthermore, the posteromedial CoA is responsible for inducing avoidance to the fox odor TMT. It is possible that the Nrp2^‐^ MCs in the dorsal OB expresses another set of targeting molecules to guide their axons to the specific CoA region to mediate aversive responses.

## 4 ACTIVITY‐DEPENDENT SYNAPSE FORMATION WITHIN THE GLOMERULI

Mammalian sensory systems are generated by a combination of activity‐dependent and ‐independent processes (Espinosa & Stryker, 2012; Hensch, 2005). The basic architecture of sensory systems is built before birth based on a genetic program. However, the neural circuits are further modified by environmental inputs. This activity‐dependent process is plastic but soon becomes irreversible. If the circuit is left unstimulated, the brain function served by that circuit becomes impaired. Thus, neonatal sensory inputs are important to make the system functional.

In the mouse olfactory system, both primary and secondary projections are genetically programmed and take place independently from each other (Inokuchi et al., 2017; Nishizumi et al., 2019). Although primary projection of OSNs autonomously occurs by axon‐axon interactions (Sakano, 2010), proper connections with M/T cells are needed in the OB to make the olfactory circuit functional. For synapse formation, association of OSN axons with the nearest neighboring M/T cells takes place and then post‐synaptic events are induced in the dendrites. Once the synapse is formed, primary dendrites of M/T cells are selected in a competitive manner and other dendrites are eliminated (Figure 7a). In contrast to the targeting of OSN axons, little is known about the events that lead to the formation of synapses with M/T‐cell dendrites. What kind of molecules mediate triggering of synapse formation, and how are the synaptic structures selected in an activity‐dependent manner?

### 4.1 Sema7A signaling induces post‐synaptic events

In the KO of CNG channels (Lin et al., 2000), both glomerular segregation and synapse formation are affected in the OB. Thus, the OR‐derived neuronal activity is required not only for the olfactory map refinement but also for synapsing OSN axons to M/T‐cell dendrites. How are the OSN axons connected firmly with the partner dendrites by OR‐derived activity? What kind of signaling molecules are essential for inducing the post‐synaptic events? To address these questions, Inoue, Nishizumi, Naritsuka, Kiyonari, and Sakano (2018) searched for a receptor and ligand pair expressed in neonates either in OSN axons or in M/T‐cell dendrites. Among more than 30 axon‐guidance, cell adhesion, and signaling molecules examined, Sema7A and its receptor PlxnC1 appeared to be promising.

Sema7A is a membrane‐bound protein localized to the pre‐synaptic axon‐terminal of OSNs. Sema7A expression is promoted by odor stimuli, but is abolished by the CNG‐channel KO or by the DRY‐motif OR mutation (Imai et al., 2006) that blocks G‐protein coupling. Thus, Sema7A expression appears to be regulated by odor‐evoked neuronal activity. It is interesting that unlike in the KOs of other Sema‐family proteins, targeting of OSN axons is not affected in the Sema7A KO. However, synapse formation and dendrite selection are perturbed in the M/T cells. Furthermore, in the KO, both pre‐ and post‐synaptic markers are markedly reduced and post‐synaptic density (PSD) is rarely formed in M/T‐cell dendrites (Inoue et al., 2018).

**Figure 9:**
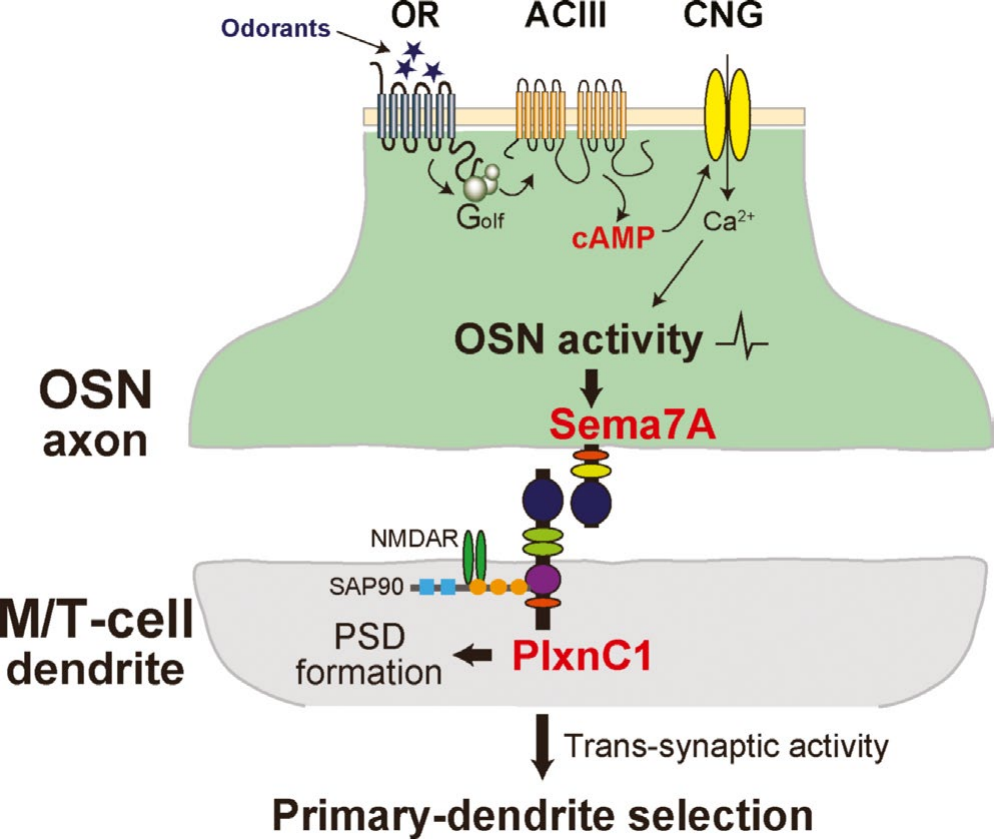
Activity‐dependent synapse formation within glomeruli. Sema7A/PlxnC1 signaling is essential for triggering post‐synaptic events in M/T cells. In OSNs, odor‐evoked OR activity produces cAMP with the aid of G_olf_ and ACIII. This cAMP opens CNG channels generating neuronal activity. Sema7A is expressed in OSN axons in an activity‐dependent manner. PlxnC1, a receptor for Sema7A, is localized to the M/T‐cell dendrites only during the first week after birth. PlxnC1, once interacts with Sema7A, recruits SAP90 for assembling the post‐synaptic density (PSD), thus promoting synapse formation. The figure is modified from Inoue and Sakano (2018)

How about PlxnC1? PlxnC1 is a receptor for Sema7A and is localized to the M/T‐cell dendrites in neonates. In contrast to Sema7A, expression of PlxnC1 is not affected by environmental odorants, but its localization to the M/T‐cell dendrites is limited to the first week after birth. In the PlxnC1 cKO specific to M/T cells, post‐synaptic events are perturbed and PSD formation is blocked as observed in the Sema7A KO. Since Sema7A and PlxnC1 are both expressed in the membrane‐bound form, it is likely that they directly interact with each other and function as signaling molecules within the glomeruli (Figure 9).

It is important to determine whether there are any additional signaling components that are regulated by OR‐derived neuronal activity for synapse formation. This can be examined by the rescue experiment in the CNG‐channel KO with the Sema7A KO background. Constitutive expression of the Tg *Sema7A* alone restored the defective phenotype of synapse formation and dendrite selection in the KO mouse. This observation demonstrates that Sema7A signaling alone is sufficient to trigger the activity‐dependent post‐synaptic events in M/T‐cell dendrites V (Inoue et al., 2018).

### 4.2 Glomerular enlargement and odor imprinting

Neonatal odor exposure promotes Sema7A expression in the responding OSNs, resulting in glomerular enlargement (Inoue et al., 2018). For example, exposure of vanillin (VNL) increases Sema7A expression in the VNL‐responsive MOR29A^+^ OSNs and promotes synapse formation and dendrite selection within the MOR29A glomeruli. Unstimulated dendrites of M/T cells that contact other glomeruli are likely removed by synaptic competition with the stimulated primary dendrite. It should be noted that these enhancements cannot be seen if the pups are exposed to VNL after the postnatal day 7 (P7).

How does this glomerular enlargement cause plastic changes in the olfactory circuit for adaptive odor responses? It has been reported that early odor exposure heightens odor‐evoked MC responses (Liu, Savya, & Urban, 2016; Liu & Urban, 2017). The enlarged glomeruli may generate stronger odor signals that are then distributed to wider areas in the OC, increasing the likelihood that new circuits are established. In the mammalian olfactory system, exposure to environmental odorants in neonates affects odor perception and behavior (Logan et al., 2012; Sullivan, Landers, Yeaman, & Wilson, 2000). As for neonatal imprinting, it is well known that ducklings follow the first moving object upon hatching, recognizing it as the parent bird (Horn, 2004; Lorenz, 1935). Although such imprinting phenomena are widely recognized, little is known about how it is established and how the imprinted memory modulates innate behavioral decisions.

Unlike other sensory systems, OSNs in mice are constantly renewed throughout the animal's life span. Although OSNs are replaced and form new connections with M/T cells, proper circuits cannot be regenerated once the existing OSNs are completely ablated after the early neonatal period. This time frame of circuit formation has been referred to as the critical period in the mouse olfactory system (Ma et al., 2014; Tsai & Barnea, 2014; Wu et al., 2018). However, the critical period has not been precisely defined at the molecular level, and the key process that makes this time‐window critical has yet to be clarified.

### 4.3 Odor imprinting during the critical period

In order to precisely determine the olfactory critical period, we recently performed unilateral naris occlusion in the newborn mice at P0. If the occluded naris is reopened after P7, synapse formation and dendrite selection are perturbed and glomerular sizes decrease in the OB. However, when occlusion is terminated at P6 or before, such impairments are not observed. These studies demonstrate that plastic changes within the glomeruli are restricted to the first week after birth, P0–7, in the mouse olfactory system (Inoue et al., submitted).

We then studied the effect of neonatal odor exposure on odor perception in adults. The mice conditioned to VNL at the early neonatal stage demonstrate increased responsiveness and sensitivity to VNL. In the preference test, the VNL‐conditioned mice spend significantly longer times in the room where a VNL‐spotted filter paper is placed. These imprinting effects cannot be seen in the mice where Sema7A signaling is blocked in neonates.

It is notable that attractive responses to imprinted odors are observed even for aversive odorants, for example, 4‐methyl‐thiazole (4MT), a derivative of fox odor TMT (Inoue et al., submitted). Stress‐reducing effects of imprinted odor memory can be seen in the odor conditioned mice. Mice usually demonstrate strong stress responses in an unfamiliar environment. When they are transferred to a new cage, the rectal temperature rises and remains elevated for approximately 20 min. However, in the mice conditioned to 4MT in neonates, stress is immediately eased by the imprinted odor as adults and a plasma concentration of the stress hormone ACTH is lowered. These stress‐reducing effects are not seen if the mice are conditioned to 4MT after the critical period.

### 4.4 Odor imprinting and social responses

Male mice normally demonstrate strong curiosity for the mouse scents of both male and female. However, the M/T‐specific cKO of PlxnC1, where Sema7A signaling is blocked, shows aversive responses by attempting to avoid interactions with unfamiliar mice (Inoue et al., submitted). Abnormal social behavior is also seen in the naris‐occluded mice, when the occlusion is performed during the entire critical period. Thus, Sema7A signaling in early neonates appears to be involved in modulating the hard‐wired circuit for smooth social interactions.

How does the imprinted odor memory induce the stress‐reducing reactions? To study this, we have analyzed various OC regions in the 4MT‐imprinted mice for their activation by the 4MT memory. Sharp contrasts are seen in the anterior MeA and AmPir between the 4MT‐conditioned and unconditioned mice (Inoue et al., submitted). By the imprinted 4MT odor, the anterior MeA is activated and the AmPir is suppressed. The anterior MeA is known to mediate attractive social responses (Inokuchi et al., 2017), whereas the AmPir induces stress reactions by raising plasma concentrations of ACTH (Kondoh et al., 2016). It has been reported that aversive odor information is transmitted to the posteromedial CoA to elicit avoidance responses (Miyamichi et al., 2011; Root et al., 2014). In the mice conditioned to 4MT, the posteromedial CoA is still activated by 4MT, however, the AmPir is suppressed. It is to be clarified how the outputs of the posteromedial CoA are blocked by the imprinted 4MT memory.

### 4.5 A possible role of oxytocin in odor imprinting

Imprinted olfactory memory always induces attractive responses to the conditional odors. How is it then that the positive quality is imposed on the neonatal odor experience? Several peptide hormones are considered as possible candidates, for example, norepinephrine, dopamine, and oxytocin, all of which are known to induce positive mental status and mediate attractive behaviors (Anderson, 2000; De Wied, Diamant, & Fodor, 1993). Among them, oxytocin appears to be promising, because oxytocin is involved in social memory formation mediated by the MeA (Gur, Tendler, & Wagner, 2014). Furthermore, oxytocin promotes attractive social interactions (Bosch & Young, 2018; Muscatelli, Desarménien, Matarazzo, & Grinevich, 2018) and is highly expressed in the neonatal brain (Sannino, Chini, & Grinevich, 2017).

To examine if oxytocin contributes to imposing the positive quality on imprinted odor memory, we have analyzed the KO mice of oxytocin (Nishimori et al., 1996) or oxytocin‐receptor (Takayanagi et al., 2005) for their odor imprinting. If conditioned to 4MT in neonates, increased responsiveness to 4MT can be seen in the both KOs. However, KO mice fail to demonstrate positive responses to the imprinted odorant 4MT and demonstrate impaired social responses (Inoue et al., submitted). When is oxytocin needed to integrate the positive quality into imprinted memory? This can be examined by rescue experiments using the pups of oxytocin KO. It was shown that if the KO pups are administrated intraperitoneally by oxytocin at P0–6, social interactions are restored in adulthood. This rescue effect is not seen when the oxytocin administration is performed after the critical period. These results indicate that oxytocin is needed in early neonates for imposing the positive quality on imprinted odor memory.

## 5 SUMMARY AND DISCUSSION

In the mammalian olfactory system, a characteristic feature of OSN projection is that both targeting and sorting of OSN axons are instructed by OR molecules (Imai et al., 2—6; Serizawa et al., 2006). Our recent studies revealed that A‐P targeting is instructed by the agonist‐independent receptor activity of ORs via protein kinase PKA, while glomerular segregation is regulated by neuronal activity via CNG channels (Nakashima et al., 2013). Furthermore, these two processes are separately regulated by distinct G‐proteins, G_s_ and G_olf_, at different stages of OSN development: G_s_ for A‐P targeting in immature OSNs and G_olf_ for local sorting in mature OSNs. Another unsolved question was how the activity is generated for A‐P targeting without odor stimuli. In the absence of agonists and inverse agonists, GPCRs freely interchange their conformations between the active and inactive forms. Baseline activity generated by this spontaneous transition is utilized for transcriptional regulation of A‐P targeting molecules.

It is generally thought that the glomerular map is used for pattern recognition of activated glomeruli to discriminate and identify odorants. During the process of OSN projection, odor information is sorted into distinct qualities and distributed to separate OB domains. Thus, the glomerular map possesses dual functions for odor detection: The map is not merely a projection screen for detected odor signals, but consists of functional domains to elicit innate odor responses. Using the optogenetic method, it was shown that once the M/T cells in a particular domain are stimulated, a specific innate behavior can be induced even through a photo‐activated single glomerulus (Saito et al., 2017).

M/T‐cell projection to the OC is important for making qualitative decisions of odor information. Particularly for innate odor responses, MC axons have to be guided properly to specific areas in the amygdala. Unlike the primary projection, secondary projection was poorly understood due to the absence of known subset markers of M/T cells. Recently, it was found that axon‐guidance molecules, Nrp2 and its repulsive ligand Sema3F, segregate MCs into two distinct subsets, Nrp2^+^ and Nrp2^‐^. Furthermore, Nrp2^+^ MCs in the posteroventral OB send their axons to the anterior MeA to mediate attractive social responses (Inokuchi et al., 2017). In contrast, Nrp2^‐^ MCs in the posterodorsal OB target the posteromedial CoA to elicit aversive responses. It is notable that Sema3F secreted by the dorsal OSN axons co‐regulates OSN targeting and MC migration along the D‐V axis. This parallel guidance is important to ensure proper matching of OSN axons and M/T cells, so that odor information is correctly transmitted from the OB to the amygdala.

During embryonic development, axon targeting of both OSNs and M/T cells takes place without involving neuronal activity. Although the basic processes of circuit formation are genetically programmed, synapses are formed in an activity‐dependent manner between the OSNs and M/T cells. Unlike in the fly and nematode, matching of periphery and projection neurons in the mouse olfactory system is not pre‐specified by the cell lineage (Nishizumi et al., 2019). Thus, the synapse formation within glomeruli is plastic and partner glomeruli for matching can be changed during evolution. This flexibility allows individuals and species to adapt to the new odor environment for their survival.

In neonates, there is a narrow time window referred to as the critical period that allows proper development of the sensory systems in response to environmental inputs. If the circuit is left unstimulated, the brain function served by that circuit becomes impaired. In the mouse, innate olfactory decisions can be modified by the neonatal odor experience. Recently, Sema7A and its receptor PlxnC1, were found to be responsible for this plastic change by triggering post‐synaptic events in the neonatal M/T cells (Inoue et al., 2018). Sema7A is expressed in the axon termini of OSNs in an activity‐dependent manner and PlxnC1 is localized to the dendrites of M/T cells only during the first week after birth, which forms the molecular basis for the olfactory critical period. Rescue experiments in the CNG‐channel KO demonstrate that Sema7A signaling is sufficient to trigger the activity‐dependent post‐synaptic events in M/T cells. In the odor‐stimulated neonatal glomeruli, primary dendrites of surrounding M/T cells are recruited. Elevated odor inputs through the enlarged glomeruli are likely responsible for odor imprinting.

Imprinting takes place by the sensory inputs during the critical period in neonates. In the mouse olfactory system, imprinted memory always induces attractive responses. The KO studies and rescue experiments indicate that oxytocin in neonates may be responsible for imposing the positive quality on imprinted memory (Inoue et al., submitted). Attractive responses can be induced even to the imprinted 4MT whose odor quality is innately aversive. Furthermore, the 4MT memory rapidly eases stress reactions by reducing the plasma concentrations of the stress hormone ACTH. Such imprinting effects can be seen only for the odor experience during the first week after birth. In the OC, imprinted 4MT memory activates the anterior MeA to mediate attractive responses and suppresses the AmPir to block the ACTH‐mediated stress reactions (Inoue et al., submitted). It is important to determine in the future how the two conflicting decisions, innate aversion and memory‐based attraction, are balanced for the imprinted 4MT.

It has been shown that early exposure to environmental odors affects social responses later in life. Male mice normally demonstrate strong curiosity toward unfamiliar mice. However, when Sema7A signaling is blocked in neonates, the mice avoid social interactions with strangers. It is possible that mice innately avoid stressful interactions with unfamiliar mice. However, Sema7A‐mediated imprinting may help pups adapt to their community for smooth social interactions with fellow mice. It will be interesting to examine whether similar imprinting described in the mouse olfactory system can be found in humans. It is also important to determine in the human infants when the critical period starts and how long it lasts. These imprinting studies will give new insights into our understanding of neurodevelopmental disorders, such as autism spectrum disorders and attachment disorders, that may be caused by improper sensory inputs during the neonatal period. The mouse olfactory system will continue to serve as a useful tool for the developmental studies of neural circuit formation in the mammalian brain.
